# A novel nanoluciferase-based system to monitor *Trypanosoma cruzi* infection in mice by bioluminescence imaging

**DOI:** 10.1371/journal.pone.0195879

**Published:** 2018-04-19

**Authors:** Erica Silberstein, Carylinda Serna, Stenio Perdigão Fragoso, Rana Nagarkatti, Alain Debrabant

**Affiliations:** 1 Laboratory of Emerging Pathogens, Center for Biologics Evaluation and Research, Food and Drug Administration, Silver Spring, Maryland, United States of America; 2 Laboratory of Molecular Biology of Trypanosomatids, Instituto Carlos Chagas/Fiocruz, Curitiba - Paraná, Brazil; Karolinska Institutet, SWEDEN

## Abstract

Chagas disease, caused by the intracellular protozoan *Trypanosoma cruzi*, affects 8–10 million people worldwide and represents a major public health challenge. There is no effective treatment or vaccine to control the disease that is characterized by a mild acute phase followed by a chronic life-long infection. Approximately 30% of chronically infected individuals develop cardiac and/or digestive pathologies. *T*. *cruzi* can invade a wide variety of nucleated cells, but only persists at specific tissues in the host. However, the mechanisms that determine tissue tropism and the progression of the infection have not been fully described. Identification of infection niches in animal models has been difficult due to the limited quantity of parasite-infected cells and their focal distribution in tissues during the chronic phase. To better understand the course of chronic infections and parasite dissemination, we developed a bioluminescence imaging system based on the use of transgenic *T*. *cruzi* Colombiana strain parasites expressing nanoluciferase. Swiss Webster mice were infected with luminescent trypomastigotes and monitored for 126 days. Whole animal *in vivo* imaging showed parasites predominantly distributed in the abdominal cavity and surrounding areas throughout the infection. Bioluminescence signal reached a peak between 14 to 21 days post infection (dpi) and decreased progressively over time. Total animal luminescence could still be measured 126 dpi while parasites remained undetectable in blood by microscopy in most animals. *Ex vivo* imaging of specific tissues and organs dissected post-mortem at 126 dpi revealed a widespread parasite distribution in the skeletal muscle, heart, intestines and mesenteric fat. Parasites were also detected in lungs and liver. This noninvasive imaging model represents a novel tool to study host-parasite interactions and to identify parasite reservoirs of chronic Chagas Disease.

## Introduction

Chagas disease (CD) is caused by infection with the parasite *Trypanosoma cruzi* and affects an estimated 8–10 million people worldwide. It has been historically considered a major public health challenge in Latin America, but international travel and changes in migration patterns are contributing to a fast globalization of the disease [1–4]. In endemic areas, transmission is mostly mediated by triatomine “kissing bug” vectors, but infections can also occur through blood transfusion, organ transplantation, ingestion of contaminated drinks and food, and from mother-to-child [5].

The initial acute stage of CD is typically distinguished by the presence of trypomastigotes in the bloodstream that can be visualized by microscopy [5]. Most infected individuals develop a cellular immune response to control the infection within 4 to 6 weeks which is not sufficient to completely eradicate the parasite, and thus progress to an asymptomatic chronic life-long infection [6]. It is estimated that approximately thirty percent of infected patients experience cardiac alterations, gastrointestinal disorders, or a combination of both, suggesting a broad range of host-parasite interactions [7].

*T*. *cruzi* uses different strategies to survive in the mammalian host such as evasion of protective immune responses, display of virulence factors and the ability to establish latency in target tissues [6]. Since the parasite can replicate in a wide variety of cell types and invade different organs, its distribution in the host is highly dynamic and associated with the progress from acute to chronic infections [8, 9].

Disease pathology and its severity could be linked to parasite persistence at specific sites [10, 11]. During the chronic phase of the disease parasites are represented mainly by amastigotes residing in a wide variety of organs including the heart, stomach and intestines, escaping immune clearance, and could possibly constitute infection reservoirs in the host [12, 13]. Adipose tissues have been also described as locations where *T*. *cruzi* may persist [14, 15]. Yet parasite distribution in tissues differs among chronically infected hosts. Recent studies conducted in animal models suggest that the parasite lineage, the genetic background of the host and the route of infection may have a significant impact on the outcome of the infection [9, 16].

Several methodologies for detection and quantification of *T*. *cruzi* in the infected host have been used, with both advantages and downsides [9]. For instance, histology allows identification of parasites *in situ* but has low sensitivity [17]. Immunohistochemistry is a more sensitive semi-quantitative method which requires careful optimization [18]. Microscopy allows quantification of blood trypomastigotes during the acute phase but does not reflect parasite burden in tissues and has poor sensitivity (~10^4^ parasites/ml) [19]. PCR-based assays are highly sensitive, but do not reflect the presence of viable parasites. In addition, only a small section of tissue or a small volume of blood is being tested by PCR, increasing the risk of false negative outcomes [9]. Overall, these approaches are limited and do not represent actual parasite localization in the host because of the focal nature of *T*. *cruzi* infection. Understanding the mechanisms that underline chronic infections may significantly contribute to the development of effective therapies to control CD. The use of real-time bioluminescence imaging has allowed successful visualization and tracking of *T*. *cruzi* parasites overtime in live infected mouse models [9, 20–23]. The sensitivity of this technology is controlled by the efficiency of visible light to travel through soft tissues, and is constantly improving with the employment of novel bioluminescence systems and more sensitive light sensors.

In this study, we describe the development of a bioluminescence imaging system that involves the use of *T*. *cruzi* Colombiana parasites expressing a novel nanoluciferase gene reporter [24], to evaluate parasite infection and tissue-specific distribution in the Swiss Webster mouse model. Non-invasive *in vivo* imaging allowed us to monitor the infection for up to 126 days. Using *ex vivo* imaging of organs and tissues, we were able to identify specific sites of parasite persistence in chronically infected mice. Our findings illustrate the nature of *T*. *cruzi* Colombiana dissemination and tissue tropism, and describe a new luminescence reporter to study CD.

## Methods

### Parasites and cells

Epimastigotes of the *T*. *cruzi* strain Colombiana (genotype TcI [25], obtained from Dr. Ester Roffe, Laboratory of Molecular Immunology, National Institute of Allergy and Infectious Diseases (NIAID), National Institutes of Health), were cultured at 26°C in LIT medium [26].

Cell monolayers of NIH/3T3 mouse fibroblast cells (ATCC^®^ CRL-1658^™^) were infected with late stationary phase epimastigotes to generate trypomastigotes. Infected NIH/3T3 cells were grown in Iscove’s Modified Dulbecco’s Media (IMDM, Invitrogen) supplemented with 5% fetal bovine serum in a humid atmosphere containing 5% CO_2_ at 37°C [27]. Alternatively, LLC-MK2 Original (ATCC^®^ CCL-7^™^) cells were used and cultured in Dulbecco′s Modified Eagle′s Medium (DMEM, Invitrogen) supplemented with 10% fetal bovine serum in a humid atmosphere containing 5% CO_2_ at 37°C.

Parasite and cell numbers were determined using a Cellometer K2 Fluorescent Viability Cell Counter (Nexcelom Bioscience, MA).

### Construct design and transfections

To obtain stable bioluminescent parasites expressing the NanoLuc^™^ luciferase (NLuc) reporter [28] we constructed a plasmid using the expression vector pBEX-v2.0 as backbone, which was generated from pBEX-GFP [29] by deletion of the GFP gene and subsequent cloning of a multiple cloning site sequence. The NLuc gene was PCR amplified from pNL1.1.CMV (Promega, MD) with forward primer NLuc F (5’-GCG GTCGACATGGTCTTCACACTCGAAGAT-3’) and reverse primer NLuc R (5’-CGCACTAGTTTACGCCAGAATGCGTTCGCAC-3’) which included *Sal*I and *Spe*I restriction sites (underlined), respectively. The resulted PCR fragment was digested with SalI and SpeI, gel purified and then inserted into the multiple cloning site using these sites, to generate the pBEX-NLuc plasmid. Nucleotide sequence and gene orientation were verified by DNA sequencing.

Transfections were performed using a total of 1 x10^7^ early-log phase wild-type epimastigotes pelleted from culture supernatants by centrifugation at 1,620 x g for 15 minutes. Parasites were resuspended in 100 μl of Human T Cell Nucleofector ^™^Solution (Lonza, Cologne), mixed with 10 μg of pBEX-NLuc and then electroporated in the Amaxa Nucleofector 2b Device, using program “U33” [21]. Transgenic parasites were obtained by selection in LIT media containing 300 μg/ml Geneticin (G418, Invitrogen, MA). Drug-resistant epimastigotes were analyzed for NLuc activity four weeks post-transfection.

### Mice and infections

The animal protocol for this study has been approved by the Institutional Animal Care and Use Committee at the Center for Biologics Evaluation and Research, US FDA (ASP 2010#03). Further, the animal protocol is in full accordance with ‘The guide for the care and use of animals’ as described in the US Public Health Service policy on Humane Care and Use of Laboratory Animals 2015 (http://grants.nih.gov/grants/olaw/references/phspolicylabanimals.pdf). All mice were maintained in the FDA/CBER AAALAC-accredited facility under standard environmental conditions for this species.

Female Swiss Webster mice were purchased from Charles River Laboratories (Germantown, MD). Mice were housed in cages under specific pathogen-free conditions and infected at 5–7 weeks of age. 5 x10^3^ cell culture derived trypomastigotes expressing NLuc, were resuspended in 0.2 ml PBS and used to infect mice via intraperitoneal (i. p.) inoculation. Peripheral blood parasitemia was determined by light microscopy examination counting the number of parasites in an unstained 5 μl of blood drawn via tail vein. The number of parasites was estimated as described [19].

### *In vitro* nanoluciferase detection assays

Epimastigotes were grown to mid-logarithmic phase in LIT media containing 300 μg/ml G418. Trypomastigotes were collected from culture supernatants of infected 3T3 cells at day 5 post-infection. Nanoluciferase activity was determined using the Nano-Glo^®^ Live Cell Assay System according to the manufacturer’s instructions (Promega, WI). Briefly, parasites were diluted to 5 x10^3^ cells per 100 μl PBS, added to a white 96-well plate and mixed with 25 μl of Nano-Glo^®^ Live Cell reagent. End-point luminescence readings were taken immediately in a Spectra Max, M5 microplate reader (Molecular Devices, PA). Alternatively, parasites were diluted to 10^5^ cells per 100 μl PBS and mixed with an equal volume of Nano-Glo^®^ Luciferase Assay Reagent, containing assay lysis buffer and furimazine substrate diluted 1/50, following the manufacturer’s recommendations. Nanoluciferase activity was determined in parasite lysates as described above.

To measure NLuc activity in intracellular amastigotes, LLC-MK2 cells were plated in 96-well plates to reach 50% confluence the next day. Triplicate wells were infected with 5 x10^4^ TcCOL-NLuc or TcCOL-wild type (wt) trypomastigotes. After an overnight incubation, the inoculum was removed and cells were washed three times with PBS. Plates were incubated for four days at 37°C in a CO_2_ incubator. Subsequently, cells were extensively washed to remove any trypomastigotes, followed by addition of 100 μl of fresh media were added per well. Finally, cells were lysed by addition of 100 μl of Nano-Glo^®^ Luciferase Assay Reagent, containing lysis buffer and furimazine (1/50 dilution). Samples were transferred to a white plate and the end-point luminescence was read in a microplate luminometer as indicated above.

To assess NLuc expression in intracellular amastigotes, LLC-MK2 cells grown to 50% confluence in borosilicate 8-well slides were infected with 5 x10^4^ TcCOL-NLuc trypomastigotes. Four days post-infection, cells were fixed with ice-cold 100% methanol for 15 minutes at -20°C and stained with 1:500 rabbit anti-NLuc antibody (Promega) followed by 1:1000 Alexa Fluor 488 conjugated anti-rabbit IgG (Invitrogen, MA). Slides were mounted with Vectashield mounting medium (Vector Laboratories, Burlingame, CA) containing 4′, 6-diamindino-2-phenylindole (DAPI). Fluorescent micrographs were acquired on a Keyene BZ-9000 fluorescence microscope at a magnification of 40X.

To establish the limit of detection achievable with the IVIS 200 system (Xenogen, CA), 100 μl of 2-fold serial dilutions of TcCOL-NLuc trypomastigotes were dispensed in triplicates in a clear 96-well plate. Next, 100 μl of Nano-Glo^®^ Luciferase Assay Reagent containing lysis buffer and furimazine (1/50 dilution) was added. The plate was immediately transferred to the IVIS 200 system camera chamber and images were taken with an exposure time of 30 seconds and medium binning. Luminescence was quantified as the sum of all detected photons counts per second (Total flux/sec) per well with the LivingImage 4.3.1 SP1 software (Xenogen, CA).

### *In vivo* bioluminescence imaging

Mice were anesthetized using 2.5% (vol/vol) gaseous isofluorane in oxygen. After anesthesia was achieved, 200 μl of a 1/20 dilution of furimazine (approximately 1 mg/Kg) in PBS [30], was injected by a single i. p. injection. Mice were then placed in the IVIS 200 system (Xenogen, CA) camera chamber, where gaseous isofluorane was administered through nose cones. Ventral images were acquired using an exposure time that varied between 30 seconds to 5 minutes depending on signal intensity. Mice were returned to their cages immediately after each imaging time point.

Data acquisition and analysis were performed by using the LivingImage 4.3.1 SP1 software (Xenogen, CA). Luminescence was quantified as the sum of all detected photons counts per second (Total flux/sec) in a chosen region of interest (ROI). The threshold bioluminescence was estimated using the mean ± 2 s.d. of the background luminescence obtained for the control uninfected mouse at each time point.

### *Ex vivo* bioluminescence imaging

Infected mice were sacrificed 126 dpi by exsanguination under terminal anesthesia and perfused with 10 ml of PBS via the heart [23]. Organs and tissues were dissected, transferred to culture/Petri dishes or 24-well plates, rinsed in PBS and soaked in a furimazine solution for 5 minutes. Images were acquired using an exposure time that varied between 30 seconds to 2 minutes depending on signal intensity. Luminescence was quantified for individual ROIs and expressed as radiance (photons/second/cm^2^/sr). Fold-changes were calculated using bioluminescence data for organs from infected mice compared with the matching organs from the uninfected mouse control. The detection threshold was estimated using the fold-change in radiance for empty ROIs determined in images taken for the infected mice compared with matching empty ROIs in images obtained for the uninfected mouse control. All tissue samples were finally washed in PBS, snap-frozen on dry ice and stored at -80°C for further evaluation.

### PCR

The integration of the pBEX-NLuc construct into the parasite genome, was verified by PCR using epimastigotes total genomic DNA as template. The Wizard Genomic DNA Purification kit (Promega, MD) was used for DNA extractions from TcCOL-NLuc and wild type control parasites. PCR reactions contained 140 ng of genomic DNA, 0.4 μM of forward primer 18 F (5’CATATGCTTGTTTCAAGGACTTAGC-3’) aligning with the 5’end of the *T*. *cruzi* Colombiana strain 18S ribosomal DNA, GeneBank accession number AF239980.1, and reverse primer NLuc R (5’-CGCACTAGTTTACGCCAGAATGCGTTCGCAC-3’) in the Platinum Taq DNA Polymerase High Fidelity mixture (Invitrogen, MA). pBEX-NLuc DNA (20 ng) was used as control. The cycling conditions were as follows: 2 minutes at 94°C, 40 cycles of 30 seconds at 94°C, 30 seconds at 54°C, 1 minute at 68°C, and a final extension of 3 minutes at 68°C. PCR fragments were resolved by agarose electrophoresis in a 1% agarose/Tris Acetate EDTA (TAE) gel stained with ethidium bromide.

DNA extractions from 25 mg of snap-frozen tissues were performed following the DNeasy Blood & Tissue Kit protocol specifications (Qiagen, MD).

Quantitative real-time PCR (qPCR) reactions were prepared using the iTaq^™^ Universal SYBR Green^®^ Supermix kit (Bio-Rad Laboratories, CA), and ran on a 7500 Fast Real-Time PCR System instrument (Thermo Fisher Scientific, MA). Each reaction contained 50 ng of DNA and 0.4 μM of *T*. *cruzi*-specific primers (Forward: 5’-AGTCGGCTGATCGTTTTCGA-3’; Reverse: 5’-AATTCCTCCAAGCAGCGGATA-3’) [27]. The genomic region encoding the IL12 p40 was used as endogenous control to verify that equal amounts of purified DNA were present in all PCR amplifications [27]. *T*. *cruzi*-specific threshold cycle (Ct) values were converted to inferred numbers of parasite equivalents by reference to a standard curve with a range of 1 x10^4^ to 1 x10^-2^ parasite equivalents. To prepare the standard curve, 1 x10^7^ TcCOL-NLuc trypomastigotes were added to a 25 mg fragment of non-infected tissue. After DNA extraction, sample’s total DNA concentration was adjusted to 50 ng/μl and serially diluted 10-fold with DNA extracted from non-infected tissue keeping a constant concentration of 50 ng/μl of total DNA per dilution.

### Enzyme Linked Aptamer (ELA) assay

ELISA plates were coated with 50 μl of plasma (1/200 dilution in PBS) from non-infected or infected mice at different times (21 to 124 dpi). After blocking with 1% bovine serum albumin (BSA), biotinylated Aptamer-29 (Apt-29) was added to each well and incubated for 1 hour at room temperature. Next, plates were washed three times with PBS and a Streptavidin-Alkaline phosphatase conjugate was added. Following a 30-minute incubation, plates were washed with PBS and the bound aptamers were detected using 4-Methyliumbeliferyl Phosphate (4-MUP, Sigma, MO). Fluorescence was read at 340 nm and emission was recorded at 440 nm, using a Spectra Max, M5 microplate reader (Molecular Devices, PA) [31].

### Statistical analysis

Data was plotted and statistical analysis was performed using GraphPad PRISM. Results are shown as means ± standard deviation. The comparison between groups was performed using unpaired t-test with a 95% confidence interval and one-way ANOVA analysis as indicated in the figure legends. Values of *p* < 0.05 were considered statistically significant.

## Results

### Transgenic *T*. *cruzi* Colombiana parasites express nanoluciferase in all life cycle stages

**Fig 1:**
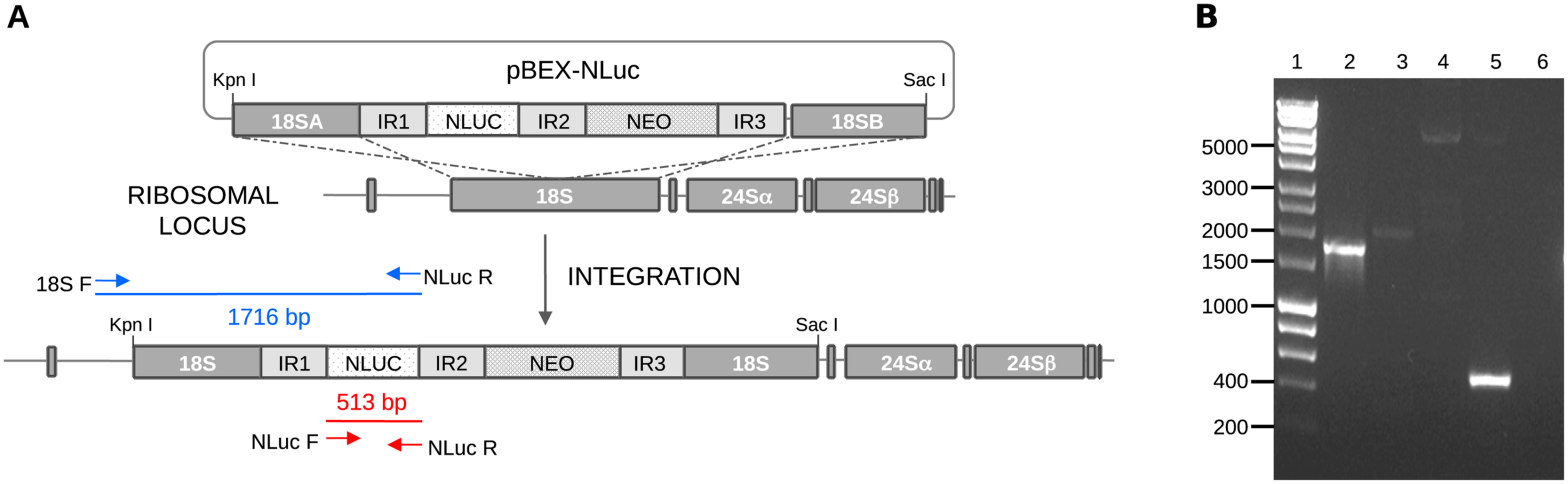
Generation of *Trypanosoma cruzi* Colombiana parasites expressing nanoluciferase. (A) Schematic representation of pBEX-NLuc and its integration into the *T*. *cruzi* ribosomal RNA gene locus by homologous recombination. 18SA and 18SB: *T*. *cruzi* 18S ribosomal RNA; IR1-3: *Strigomonas culicis* tubulin intergenic regions [29]; NLUC: nanoluciferase gene; NEO: neomycin phospotransferase gene. (B) PCR amplification of the 18SA fragment followed by the IR1 and NLuc sequences was performed using genomic DNA extracted from TcCOL-NLuc and primers 18SF and NLuc R (lane 2). Genomic DNA extracted from TcCOL-wt and DNA from pBEX-NLuc with identical set of primers, were included as negative controls (lanes 3 and 4, respectively). The NLuc gene was amplified from pBEX-NLuc using primers NLuc F and NLuc R and used as positive control (lane 5). PCR products were evaluated in a 1% agarose gel stained with ethidium bromide. 1 Kb ladder (lane 1); non-template PCR control (lane 6).

We engineered stable bioluminescent Colombiana strain [25] epimastigotes, by integrating the NLuc gene [28] into the 18S ribosomal DNA parasite gene locus. Briefly, a PCR fragment coding for NLuc was cloned into the *Spe*I and *Sal*I sites of pBEX-v2.0 [29] to obtain a recombinant plasmid termed pBEX-NLuc (Fig 1A).

The new construct contains the *Strigomonas culicis* tubulin intergenic regions (IR1-3) flanking the NLuc and the selectable marker neomycin phosphotransferase (NEO) genes for correct transcript processing [29], intercalated into the 18S ribosomal RNA (Fig 1A, 18SA and 18SB) sequences used for targeted integration.

Early-log phase wild-type epimastigotes were transfected with pBEX-NLuc and then grown in G418-supplemented medium to select cells containing the integrated construct. The integration of the NLuc and NEO genes into the parasite genome was demonstrated by PCR using total genomic DNA isolated from drug resistant epimastigotes. We designed primer 18S F that covers nucleotides 1 to 25 of the *T*. *cruzi* Colombiana strain 18S rDNA (GeneBank accession number AF239980.1), a region that is not included in the plasmid [29]. A single PCR fragment of 1716 bp (Fig 1B, lane 2) was amplified from TcCOL-NLuc genomic DNA using primers 18S F and NLuc R (Fig 1A). This fragment was not amplified when using TcCOL-wt genomic DNA as the wt parasites do not have the NLuc gene (Fig 1B, lane 3). Likewise, no amplification was detected when using pBEX-NLuc plasmid and the same set of primers, since nucleotides 1–25 of the 18S rDNA were not present in the construct (Fig 1B, lane 4). A reaction containing the set of primers NLuc F/Nluc R and pBEX-NLuc, rendered a single PCR product of 513 bp and served as a positive control for this experiment (Fig 1B, lane 5). The PCR analysis results indicate that the NLuc and NEO genes were integrated in the predicted site of the parasite ribosomal RNA gene locus.

**Fig 2:**
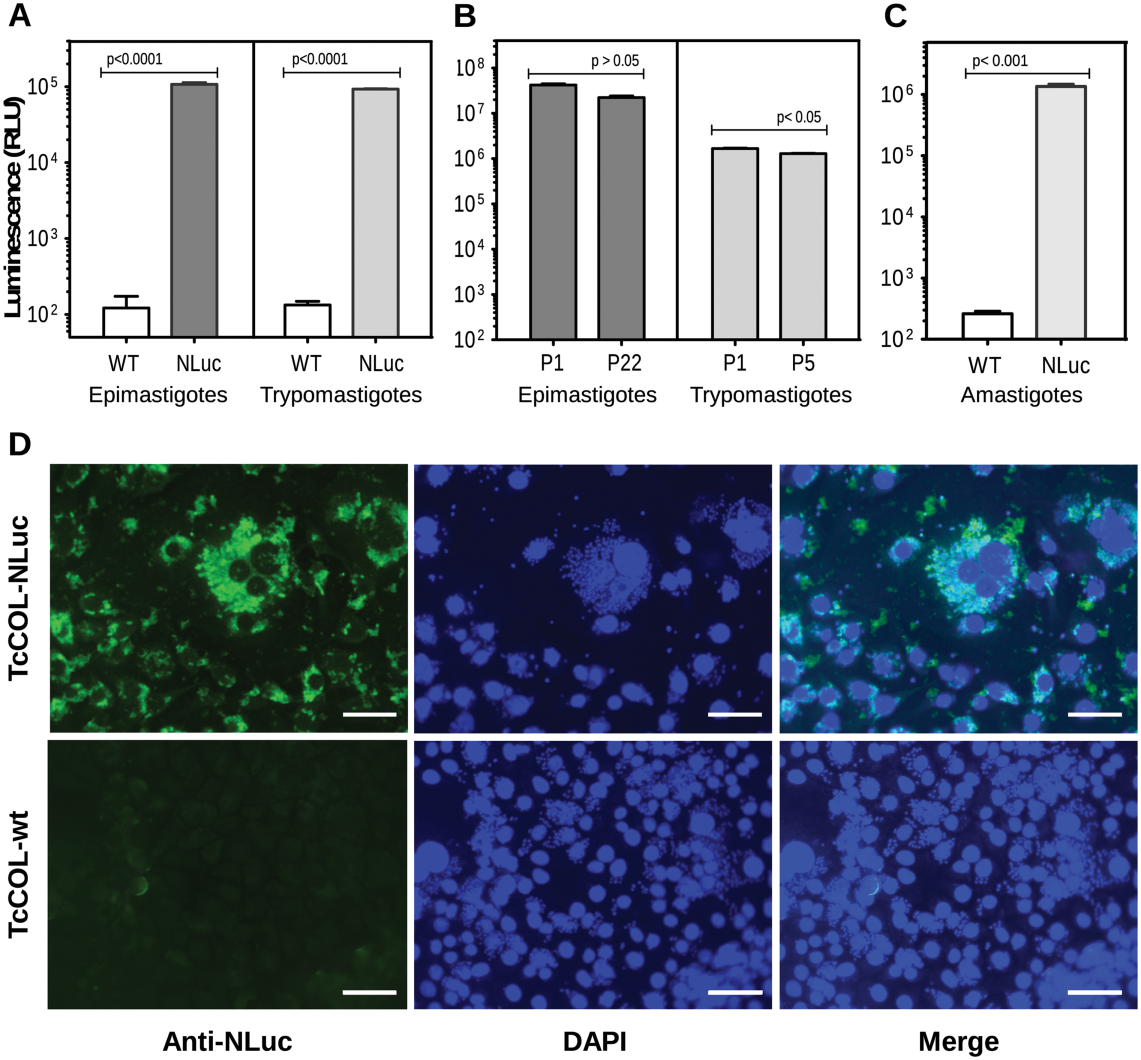
Expression of nanoluciferase in transgenic TcCOL-NLuc parasites. (A) Luciferase activity was measured in 5 x10^3^ live TcCOL-NLuc and TcCOL-wt cells using the Nano-Glo^®^ Live Cell Assay System. (B) Luciferase activity was measured in lysates of 10^5^ TcCOL-NLuc and TcCOL-wt cells using the Nano-Glo^®^ Luciferase Assay System. Left panel: NLucexpression evaluated in TcCOL-NLuc epimastigotes after passage 1 (P1) and passage 22 (P22) in LIT media containing G418. Right panel: NLuc expression evaluated in tissue culture-derived TcCOL-NLuc trypomastigotes after passage 1 (P1) and passage 5 (P5) in LLC-MK2 cells. (C) Luciferase activity in amastigotes was evaluated in LLC-MK2 cells infected with 5 x10^4^ TcCOL-NLuc or TcCOL-wt trypomastigotes. Four days post-infection, luminescence was assessed in cell lysates using the Nano-Glo^®^ Luciferase Assay System. (D) Nanoluciferase protein expression in intracellular amastigotes was evaluated in methanol-fixed LLC-MK2 cells infected with 5 x10^4^ TcCOL-NLuc (top panels) or TcCOL-wt (bottom panels) trypomastigotes by immunostaining with rabbit anti-NLuc antibodies (Anti-NLuc). DAPI indicates DNA staining. Values in (A), (B) and (C) represent means ± s.d. of three replicate wells. Statistical differences between groups were calculated using an unpaired t-test with a 95% confidence interval. *p*-values are indicated. Scale bar: 50 μm.

Four weeks post-transfection, G418-resistant epimastigotes displayed NLuc activity (Fig 2A) indicating successful expression of the integrated NLuc gene.

Luminescence remained stable in epimastigotes for up to 22 *in vitro* passages in the presence of G418 (Fig 2B). Stationary phase epimastigotes expressing NLuc were capable of infecting naïve NIH/3T3 cells. NLuc reporter activity was detected in trypomastigotes collected from infected NIH/3T3 cells (Fig 2A). The NLuc activity in trypomastigotes diminished slightly but significantly (*p* < 0.05) through the 5 consecutive passages tested in this experiment (Fig 2B). NLuc activity was also detected in lysates of LLC-MK2 cells infected (Fig 2C). Immunostaining of TcCOL-NLuc-infected MK2 cells with anti-NLuc antibodies, revealed NLuc protein expression in intracellular amastigotes (Fig 2D, top panels). In contrast, no immunostaining with anti-NLuc antibodies was detected in cells infected with TcCOL-wt trypomastigotes (Fig 2D, bottom panels).

Together, the results illustrated in Fig 2 show that the genetically modified parasites express NLuc activity throughout the three developmental stages, and that they can complete a full life cycle *in vitro* as the parental wild-type strain.

### *In vivo* imaging with bioluminescent *T*. *cruzi* Colombiana trypomastigotes represents a sensitive noninvasive tool to monitor parasite infection in Swiss Webster mice

**Fig 3:**
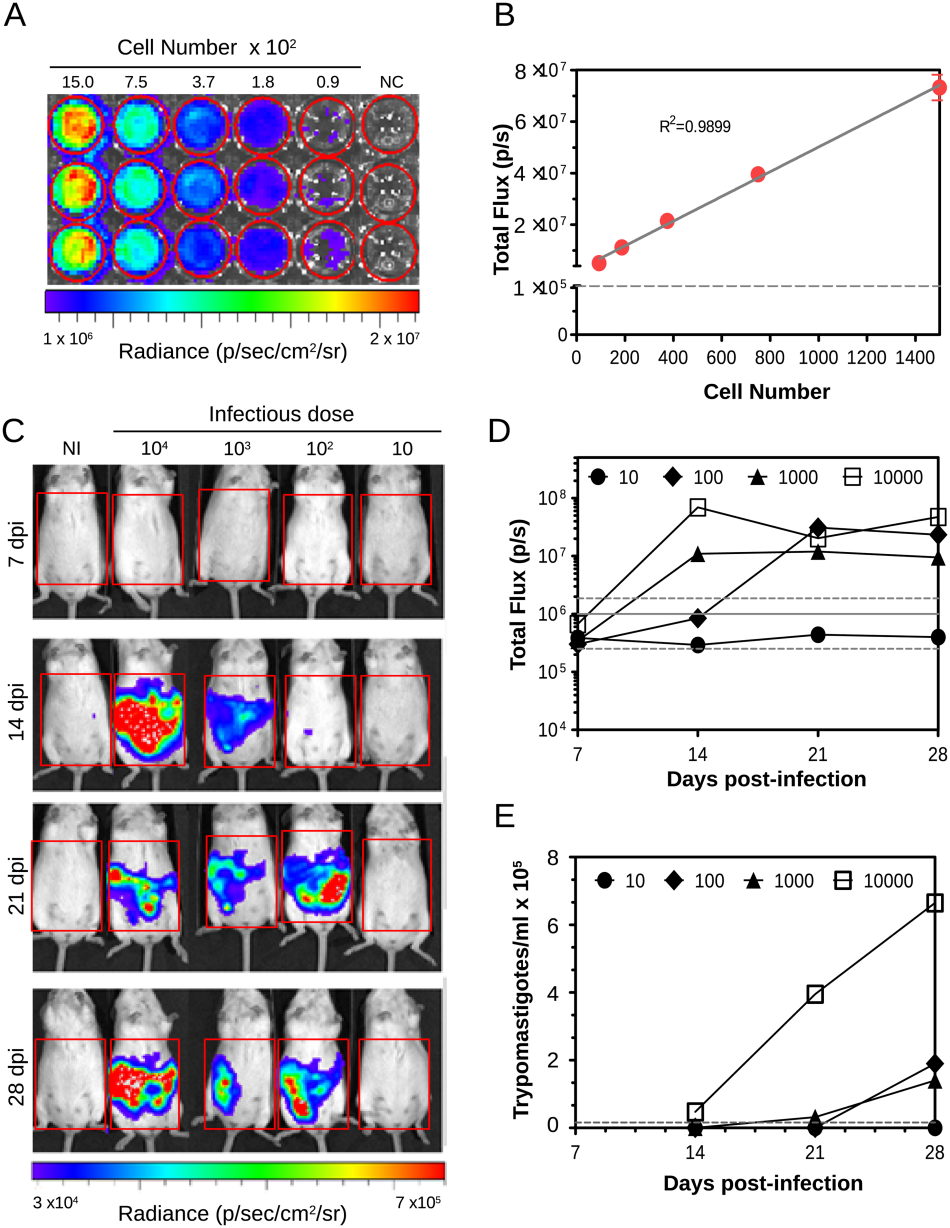
Live bioluminescence imaging of Swiss Webster mice infected with increasing doses of TcCOL-Nluc parasites during the acute phase. (A) Image of a 96-well plate containing 100 μl of 2-fold serial dilutions of TcCOL-NLuc trypomastigotes. After addition of the Nano-Glo^®^ Luciferase Assay, the plate was transferred to the IVIS 200 system camera chamber and imaged with an exposure time of 30 seconds and medium binning. (B) Linear regression plot generated using luminescence values (total flux expressed as photons/second) recorded for each well. Data represent means ± s. d. of three replicate wells. The dotted line indicates the background luminescence of the control wells. (C) Ventral view images of mice infected with increasing numbers of TcCOL-Nluc tissue culture-derived trypomastigotes (10 to 10^4^ parasites per dose) taken at 7, 14, 21 and 28 dpi. (D) Bioluminescence signal (total flux) measured over time for each animal in the regions of interest indicated by red rectangles in A, and expressed as total flux (photons/sec). Grey lines indicate the detection threshold determined as the mean (solid line) and mean ±2 s.d. (dashed lines) of background luminescence of the control uninfected mouse. (E) Blood parasitemia (trypomastigotes/ml) quantified by microscopy at the indicated times post-infection. Grey dotted line represents the limit of detection (1.58 x10^4^ parasites/ml blood). Log _10_ heat-map scales represent bioluminescence intensity (blue: low; red: high). NI: non-infected mouse.

We first analyzed the capability of the IVIS 200 imaging system to detect luminescent parasites *in vitro*. Serial dilutions of TcCOL-NLuc trypomastigotes were plated in a 96-well plate and images were acquired after addition of the Nano-Glo^®^ Luciferase Assay Reagent. Bioluminescence intensity decreased with the number of cells (Fig 3A), with an estimated limit of detection of approximately 100 parasites. A significant correlation between signal strength expressed as total flux (photons/sec) and parasite number was found using linear regression analysis (R^2^ = 0.9899, Fig 3B).

Next, we assessed the infectivity of bioluminescent *T*. *cruzi* Colombiana trypomastigotes in Swiss Webster mice. This experimental CD model leads to a well characterized chronic infection [32] and was successfully used in our laboratory to study CD biomarkers [27].

A pilot experiment was conducted to determine the most suitable delivery route of the furimazine substrate (S1 Fig). Briefly, Swiss Webster mice were infected by intraperitoneal (i. p.) injection with 5 x10^3^ tissue culture-derived TcCOL-Nluc trypomastigotes. A mock-infected mouse served as negative control for background determination. Twenty one days post-infection, animals were injected with furimazine via the tail vein (intravenous injection, i. v.) or in the peritoneal cavity (i. p.), anesthetized and transferred to the IVIS 200 system. *In vivo* imaging showed bioluminescence mainly in the abdominal cavity after either i. p. (S1A Fig, top panel) or i. v. (S1A Fig, bottom panel) delivery of the substrate. We found no significant differences when we measured the total ventral bioluminescence intensity (S1B Fig). Based on our experimental observations, and previous reports [33, 34] describing the successful delivery of furimazine in the peritoneal cavity, the i. p. route was the method of choice in all subsequent experiments.

To study the effect of the *T*. *cruzi* infectious dose, animals were inoculated i. p. with different amounts (10 to 10^4^ parasites) of TcCOL-NLuc tissue culture-derived trypomastigotes. *In vivo* images were taken at 7, 14, 21 and 28 dpi, according to the procedures described in materials and methods. At each time point, mice were injected with furimazine, anesthetized, and ventral images were taken using the IVIS 200 system. Bioluminescence intensity was measured in regions of interest using the LivingImage software. Peripheral blood parasitemia was determined by microscopic examination [19].

Real-time imaging allowed the visualization of infection in the abdominal cavity with the signal increasing over time for all parasite doses except for the mouse that was infected with 10 parasites and the mock-infected control (Fig 3C).

The peak luminescence reached at 14 dpi when using either 10^4^ or 10^3^ parasites, and at 21 dpi in the mouse infected with 10^2^ parasites (Fig 3D). In addition, 3 weeks post-infection, signal intensities (total flux) became independent of the parasite dose used for initial infection, reaching almost identical levels among the animals. Parasites became detectable in peripheral blood by microscopy 14 dpi with a 10^4^ infection dose, and 21 dpi when animals were inoculated with 10^3^ parasites while with a 10^2^ infection dose, parasites could be detected in blood only starting at 28 dpi (Fig 3E).

Our results demonstrate that this *in vivo* imaging system can be effectively used to follow *T*. *cruzi* infection through the acute phase. Furthermore, it may be successfully used for parasite detection before peripheral blood parasitemia could be measured by microscopy (Fig 3D and 3E).

### Infection of Swiss Webster mice with luminescent T*rypanosoma cruzi* Colombiana trypomastigotes can be tracked throughout the chronic phase by live bioluminescence imaging

To better understand the progression of *T*. *cruzi* Colombiana infection, a group of seven Swiss Webster mice were infected i. p. with 5 x10^3^ tissue culture-derived TcCOL-NLuc trypomastigotes. A mock-infected mouse served as negative control for background determination. At different times post-infection (from 7 to 126 dpi), mice were analyzed by *in vivo* imaging as described above. The course of infection was also monitored in blood samples (collected at the same times post-infection) by microscopy, qPCR, and by Enzyme Linked Aptamer (ELA) assay, an aptamer-based method which allows detection of *T*. *cruzi* excreted secreted antigens (TESA biomarkers) in blood of infected mice [27, 31].

**Fig 4:**
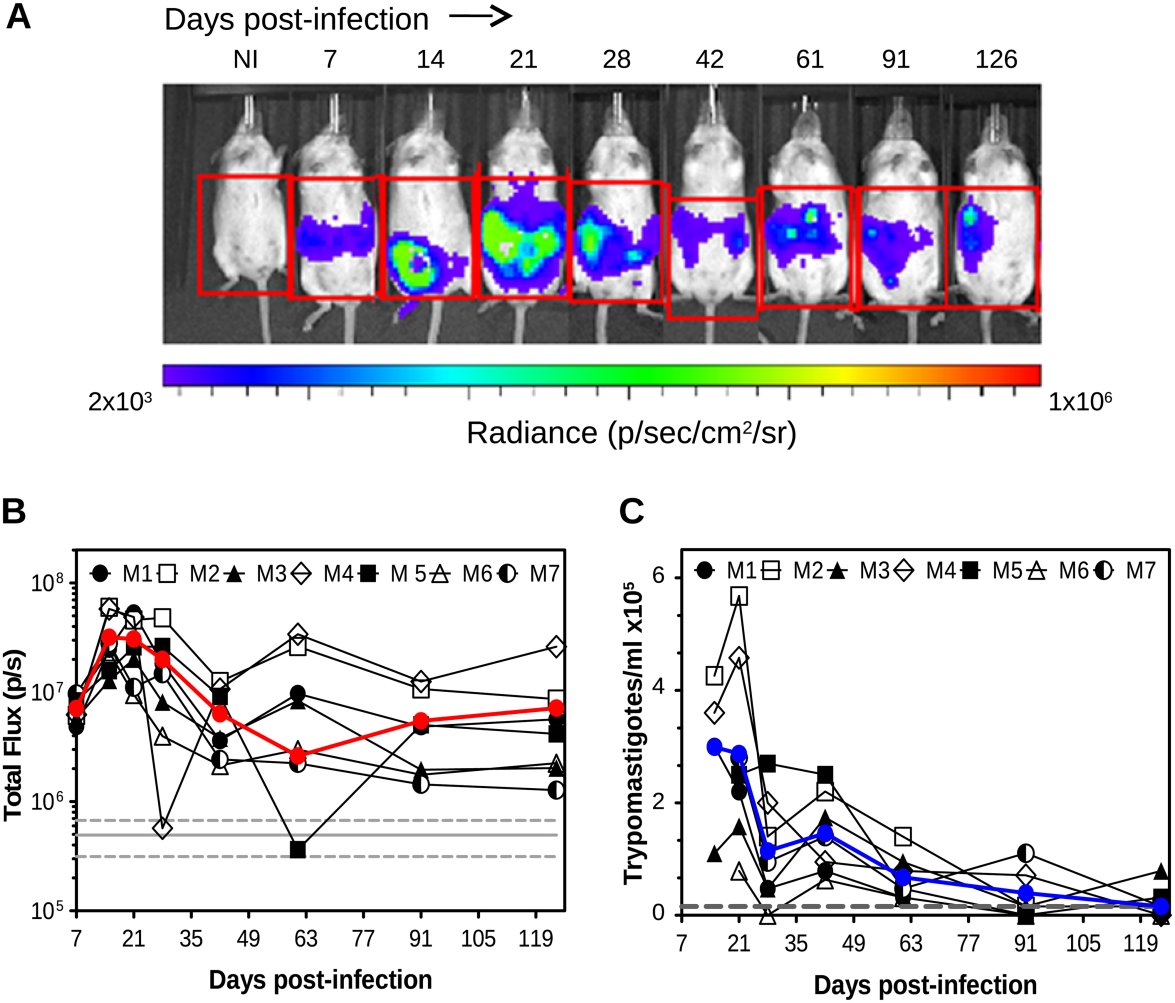
Monitoring the course of TcCOL-NLuc chronic infection in Swiss Webster mice by live bioluminescence imaging. (A) Representative ventral view images of mouse #1 infected with TcCOL-NLuc tissue culture-derived trypomastigotes (5 x10^3^ parasites) taken at the indicated time points. NI: non-infected control mouse. Log _10_ heat-map scale represents bioluminescence intensity (blue: low; red: high). (B) Total abdominal bioluminescence (total flux) for seven individual mice and their mean (red line) measured in the regions of interest indicated in (A) and over the course of infection. The grey line indicates the detection threshold determined as the mean (solid line) and mean ± 2 s.d. (dashed lines) of background luminescence of the control uninfected mouse. (C) Blood parasitemia (trypomastigotes/ml) determined by microscopy over time for each individual mouse and mean (blue line). Grey line indicates the limit of detection (1.58 x10^4^ parasites/ml blood).

Representative images of the same animal taken over a period of 126 dpi are displayed in Fig 4A.

The bioluminescence distribution followed a similar pattern in all seven mice, showing parasites disseminated and typically localized in the abdominal cavity, although there were some variations in the position of infected foci between time points. In this experiment, quantification of ventral luminescence was possible as early as 7 dpi (Fig 4B). The peak of bioluminescence was reached between 14 and 21 dpi with variations among infected animals (Fig 4B). The signal decreased progressively over time, but could still be measured at 126 dpi in all 7 animals. Bioluminescence remained detectable in one of the infected animals that we followed up for 222 days (S2 Fig). In contrast, the number of parasites present in blood showed a peak at 21 dpi in all seven infected mice (Fig 4C), and gradually decreased to levels close to the limit of detection of the microscopy-based quantification. Blood parasitemia was not detectable in 3/7 mice at 126 dpi. Additionally, the infection status of all seven mice was verified using the ELA assay and circulating biomarkers could be identified in serum samples from 21 to 126 dpi (S3 Fig).

These live imaging-based studies demonstrate the potential use of TcCOL-NLuc/Swiss Webster mouse model to evaluate parasite distribution and infection progression until the chronic stage of CD. Nevertheless, association between whole animal bioluminescence and infection of specific tissues and organs can’t be accomplished using this live imaging approach.

### *Ex vivo* imaging of tissues and organs isolated from mice chronically infected with luminescent *T*. *cruzi* Colombiana parasites reveals sites of parasite persistence

To further evaluate the distribution of bioluminescent parasites in various tissues and organs we performed *ex vivo* imaging of mice infected with TcCOL-NLuc. Briefly, animals were sacrificed 126 dpi by exsanguination under terminal anesthesia and perfused with 10 ml of PBS via the heart to eliminate the blood trypomastigotes from the animal [23]. Organs and tissues were dissected, washed in PBS and transferred to 24-well plates or Petri dishes where they were incubated with furimazine for 5 minutes.

**Fig 5:**
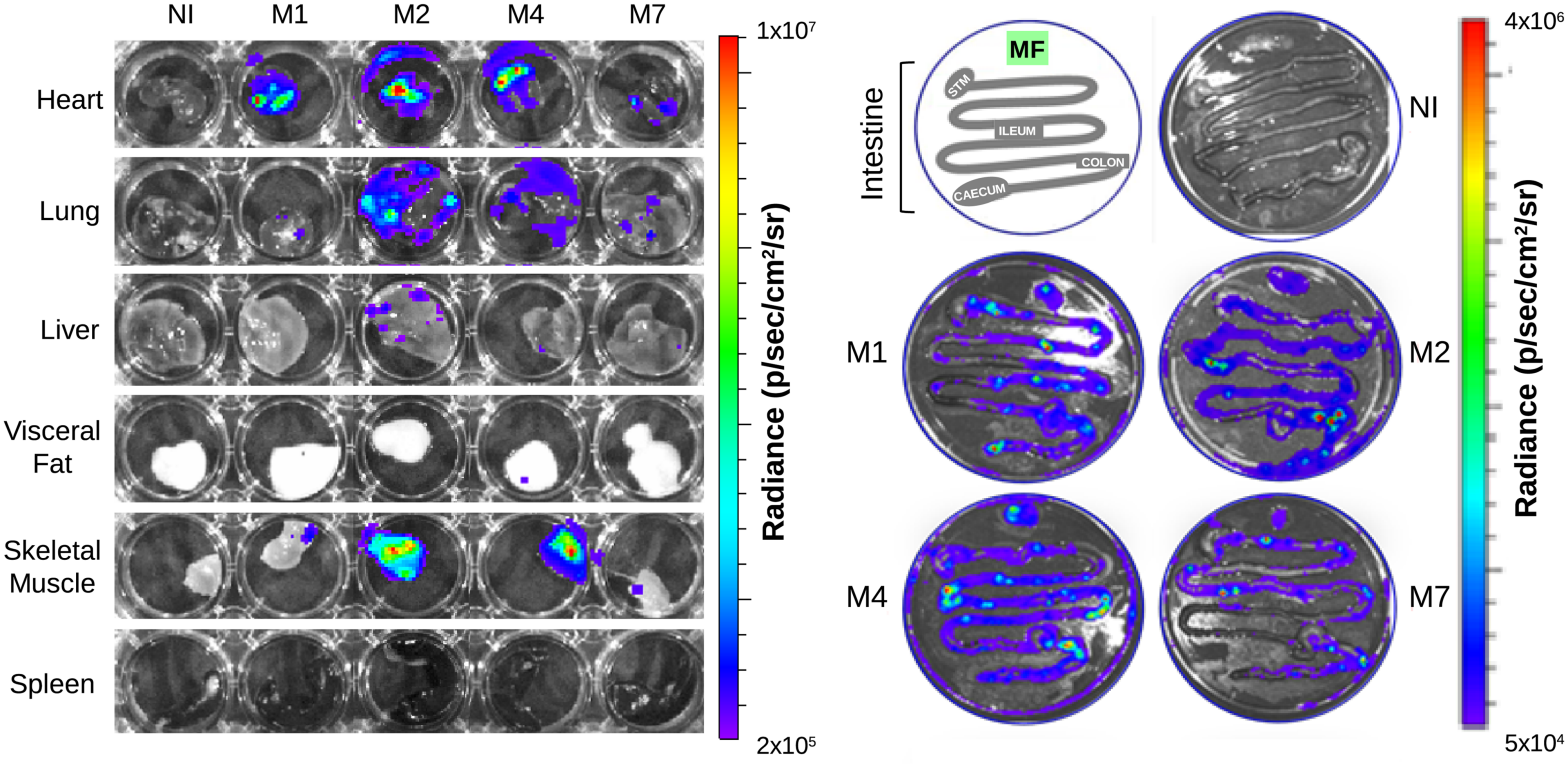
*Ex vivo* bioluminescence imaging of selected organs and tissue samples imaged at 126 dpi with TcCOL-NLuc. Four randomly selected TcCOL-NLuc-infected mice (M1, M2, M4, and M7) and one non-infected mouse control (NI) were sacrificed 126 dpi. Organs and tissues were excised and transferred to a 24-well plate or Petri dishes. After rinsing with PBS, samples were soaked in a furimazine solution for 5 minutes. Images were acquired using an exposure time from 30 seconds to 2 minutes. Log _10_ heat-map scales represent bioluminescence intensity (blue: low; red: high). MF: mesenteric fat.

Examination of excised tissues and organs by bioluminescence imaging allowed identification of TcCOL-NLuc parasites mainly in the heart, skeletal muscle, lung, liver, mesenteric fat, and intestine (Fig 5).

No luminescence signal could be detected in the spleen. However, the spleens of the chronically infected mice were approximately three times (Mean = 0.3 g) the mass of the uninfected control mouse (0.1 g), a typical observation in experimental *T*. *cruzi* infections [35, 36].

**Fig 6:**
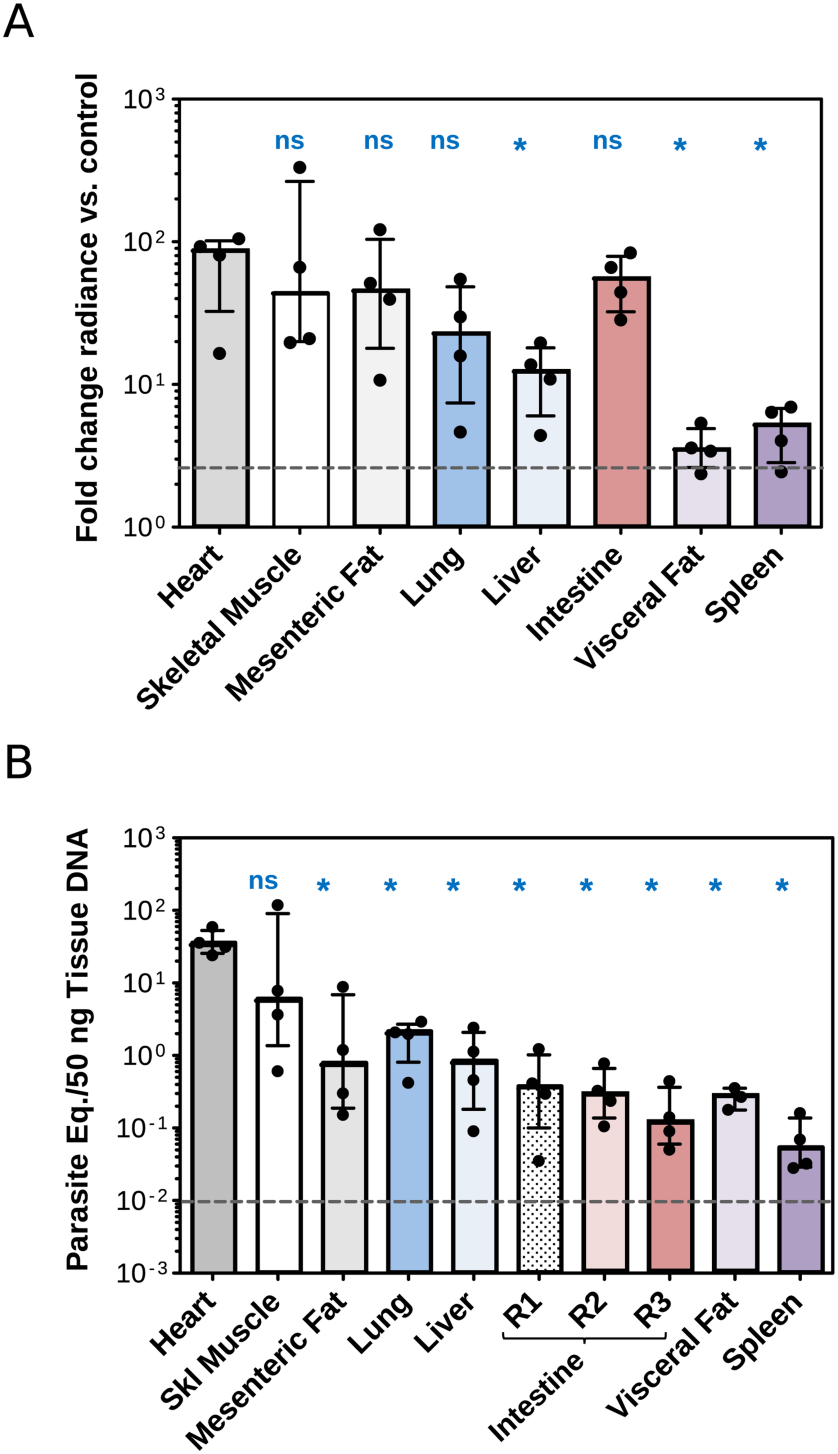
Quantification of parasite loads in tissues and organs of chronically TcCOL-NLuc-infected mice at 126 dpi by bioluminescence and qPCR. (A) Luminescence signal quantified for each individual organ/tissue shown in Fig 5 and expressed as radiance (photons/second/cm^2^/sr). Data represent fold-changes in bioluminescence intensity for organs from infected mice compared to the matching organ from the non-infected control mouse. The detection threshold (grey dashed line) was estimated using the fold-change in radiance for an empty region determined in images taken for the infected mice compared with a matching empty region in images obtained for the infected mouse control. (B) qPCR evaluation of tissue-specific parasite loads. Reactions were conducted using 50 ng of tissue DNA isolated from four TcCOL-NLuc- infected mice and one non-infected mouse. *T*. *cruzi*-specific threshold cycle (Ct) values were converted to inferred numbers of parasite equivalents by reference to a standard curve with a range of 1 x10^4^ to 1 x10^-2^ parasite equivalents/50 ng of tissue DNA. The IL12 p40 genomic region was used as endogenous control to verify that equal amounts of purified DNA were present in all PCR amplifications. Grey dashed lines indicate the limit of detection (1 x10^-2^ parasite equivalents). Data represent the median with interquartile range. Statistical differences among tissues were calculated using one-way ANOVA analysis in GraphPad Prism. *: *p* < 0.05 indicates significant difference with the heart; ns: no significant difference with the heart.

To quantitate the bioluminescence signal in the tissues and organs, individual regions of interest were delineated for each specimen and the luminescence was measured for these regions using the LivingImage software. Results were expressed as fold-changes in radiance (photons/sec/cm^2^/sr) as described in the materials and methods section, and denote parasite density per tissue/organ (Fig 6A).

Because the Colombiana strain of *T*. *cruzi* was shown to have tropism to the myocardium in mice [32, 37], we used the heart as reference tissue in our analysis. The fold change values calculated for the skeletal muscle, lungs, intestines and mesenteric fat were not significantly different than those obtained for the heart. However, we identified significantly lower fold change of luminescence signals in the, liver, spleen and visceral fat compared to the heart (Fig 5 and S1 Table).

Finally, parasite loads were determined in collected tissues and organs using tissue-specific qPCRs to quantify parasite DNA (Fig 6B). *T*. *cruzi* DNA standard curves were prepared for each organ (with a linear range of 1 x10^-2^ to 1 x10^4^ parasite equivalents per 50 ng of tissue DNA). A representative curve for the heart is illustrated in S4 Fig. As expected based on our bioluminescence results, the heart showed significantly higher parasite loads (median of 33.31 parasite equivalents/50 ng heart DNA) followed by the skeletal muscle, lung, liver, mesenteric fat, intestine, visceral fat and spleen, with 5.71, 1.96, 0.79, 0.74, 0.25, 0.22 and 0.05 median of parasite equivalents/50ng tissue DNA, respectively (Fig 6B and S2 Table). The apparent low parasite loads found in 3 regions of the intestine (R1-R3) could be attributed to the fact that we analyzed randomly excised sections of the small intestine. Previous findings [38] revealed that the small intestine of mice experimentally injected with two Mexican TcI *T*. *cruzi* strains, was infected to a lesser degree than the colon. It should also be noted that the fold change in radiance was calculated including the full area covered by the intestine as the region of interest. This observation clearly reflects the bias nature of using qPCR that relies on the study of a small section of tissue and fails to evaluate parasite load in the entire organ. In addition, parasites were undetectable in the visceral fat sample obtained from one animal (Fig 6B) that showed parasite equivalent levels significantly lower than the limit of detection of the qPCR (10^−2^ parasite equivalent per 50 ng of tissue DNA).

Taken together, these experiments revealed a widespread parasite distribution in our animal model, in which the skeletal muscle, heart, intestines and mesenteric fat appear to be preferred infection sites. Our results also indicate that the lung, liver, spleen and visceral fat remain infected during a chronic infection with *T*. *cruzi* Colombiana parasites.

## Discussion

Little is known about the mechanisms that regulate infection of host cells by *T*. *cruzi* and what determines the development of cardiomyopathy, megacolon, and megaesophagus or a combination of these pathologies [6, 39]. Data suggest that an ongoing infection is needed to cause tissue damage [10, 11] while complex host-parasite interactions may govern the ability of the parasite to persist in preferential sites during the chronic phase. Evasion from the host immune response, parasite’s genetic background and capacity to invade multiple organs, may influence the different clinical manifestations observed in patients. While the use of experimental mice models has contributed to define many aspects of the disease, more investigation is required to develop suitable *in vivo* tools to better understand the progression of the infection and accurately reveal sites of parasite persistence.

In the past several years, the use of bioluminescence techniques facilitated the analysis of parasite distribution within small animals in real time, without requiring animal euthanasia to visualize infected tissues. The fate of luminescent *T*. *cruzi* parasites belonging to different lineages and expressing a variety of luciferase reporters was successfully followed over the course of acute and chronic infections in several mouse strains [9, 20, 40]. Here, we report a new model to characterize a *T*. *cruzi* infection, using the Colombiana strain expressing nanoluciferase (NLuc). The protein is a novel luminescence reporter that has been shown to offer several advantages over other luciferases, such as smaller size (19 kDa vs. 60 kDa for firefly luciferase), improved stability, and increased luminescence emission [24, 28]. This approach to monitor *T*. *cruzi* infection constitutes the first description of a mouse model that combines the *T*. *cruzi* Colombiana strain expressing NLuc and Swiss Webster mice. We show that the genetically modified TcCOL-NLuc parasites expressed NLuc in all three life cycle stages, and that luminescence was stable after several *in vitro* passages with and without sustained drug pressure (Fig 2A and 2B). In our studies, we did not investigate the role played by the pBEX-NLuc plasmid construct itself in the overall expression of NLuc activity. The regions flanking the NLuc gene in this plasmid construct are different from those used in other recent studies [16, 23] and could possible play a role in the efficient expression of the NLuc gene. However, the limit of detection that we achieved by imaging serial dilutions of NLuc-expressing trypomastigotes with the IVIS 200 system, was approximately 100 parasites, which is comparable to previously published reports [23, 41].

By infecting Swiss Webster mice, we could monitor the infection by *in vivo* imaging from 7 to 126 dpi (Figs 3 and 4) and were able to identify specific sites of parasite persistence in chronically infected mice by *ex vivo* imaging of dissected tissues and organs at 126 dpi (Figs 5 and 6A). We found that in live whole body imaging, the bioluminescence was detected in the abdomen and surrounding areas throughout the infection. The signal intensity progressively decreased as the animals reached the chronic phase, reflecting the progressive reduction in the overall parasite burden over time. Interestingly, we could visualize luminescent parasites in the acute phase using *in vivo* imaging before they could be detected in blood by microscopy (21 dpi), even in a mouse that was inoculated with a low infection dose of 100 parasites (Fig 3). Our data are consistent with previous reports showing that *T*. *cruzi* remained easily detectable by bioluminescence when peripheral blood sample counts were below the microscopy limit of detection observation [23]. Likewise, the higher sensitivity of detection achieved using bioluminescent parasites compared to blood microscopy was also observed when we followed seven animals through the chronic phase. The luminescence signal remained stable after 63 dpi till the end of the experiment (126 dpi), while the number of parasites in blood decreased over time to undetectable levels (Fig 4B and 4C).

Nanoluciferase is a relatively new luciferase compared to the widely used firefly, *Renilla* or *Gaussia* luciferases and its application for *in vivo* imaging remains to be fully investigated [24, 28]. To date, NLuc and its furimazine substrate have been effectively employed as bioluminescence system to study spread and tissue tropism of pathogens in real time imaging [23, 34, 42, 43] with light signal outputs greater than that of *Gaussia* and firefly luciferases [30]. However, the use of NLuc to track *T*. *cruzi* dissemination in animal models has not been documented before. Although the bright signal and low background of the reporter and substrate combination may provide higher sensitivity in living animals, luminescence detection in deep tissues could be limited due to the emission wavelength of NLuc at approximately 460 nm compared to greater than 600 nm for the red-shifted luciferase which provides reduced tissue absorbance [24]. Further studies to compare side-by-side NLuc with other luminescence reporters will be critical to determine the advantages (if any) of these NLuc-expressing *T*. *cruzi* parasites to identify tissues of low parasite load in chronically infected mice using live imaging.

Chagas Disease is associated with *T*. *cruzi* persistent infection [10, 11]. Several reports have shown that parasites can persist in tissues of infected experimental animals [10, 14, 23] and humans [44–46]. Remarkably, tissue tropism is influenced by the parasite strain [47] and host genetic background [9], leading to a differential distribution among chronically infected hosts. For instance, the highly resistant to chemotherapy Colombiana strain of *T*. *cruzi* [48, 49] is characterized by a predominant tropism to skeletal muscle and myocardium in mice [32, 37]. Our *ex vivo* bioluminescence imaging experiments showed the presence of Tc-COL-NLuc parasites in the heart, skeletal muscle (isolated from quadriceps), lung, liver, mesenteric fat and intestine but not in the spleen (Figs 5 and 6A). These results correlate with a recently published report that visualized *T*. *cruzi* Colombiana strain amastigote nests in skeletal muscle myocytes using histopathological analysis [32]. Similarly, previous imaging studies in BALB/c and C57BL/6 mice, infected with luminescent *T*. *cruzi* genotype Tc I Dm28c and JR strains, showed a tissue distribution comparable to the Colombiana strain, with mainly gastrointestinal persistence and heart infection [9, 40]. It should be noted that bioluminescence imaging lacks the ability to detect parasites that are metabolically inactive, and consequently, do not produce NLuc [9, 21]. Yet, the presence of dormant *T*. *cruzi* parasites in infected tissues has not been reported.

Our quantitative PCR data support the presence of parasite-specific DNA in most of the tissues that were analyzed (Fig 6B), which is in agreement with recently published studies [32]. We found low parasite loads in 3 different regions excised from the small intestine (Fig 6B, R1-R3). This contrasts with a high luminescence signal observed in the entire organ using *ex vivo* imaging. However, the luminescence imaging results suggest that the parasites are not evenly distributed among the entire length of the intestine which shows scattered small areas of higher parasite content with radiance of 1 x10^6^ p/sec/cm2/sr (Fig 5). We believe that the 3 sections analyzed by qPCR have low parasite loads and may not be representative of the whole intestine. Furthermore, there is evidence in the literature that the small intestine may be less infected than the colon in *T*. *cruzi* infected mice [38], which could also explain the discrepancy observed for this tissue.

A bright luminescence signal was observed *ex vivo* in hearts of mice that were sacrificed at 22 dpi (S1 Fig). Hearts harvested at 126 dpi also showed a significant number of parasites by both *ex vivo* imaging and qPCR analyses (Figs 5 and 6). Surprisingly, there was no bioluminescence associated with the expected position of the heart at the same time points using real-time imaging (Figs 3 and 4; S1 and S2 Figs). Because the luminescence signal distribution was independent of the route of furimazine delivery (S1 Fig) and assuming that the substrate reached the heart when injected i. v., we believe that the failure to identify infected hearts by real-time bioluminescence is likely due to the limitation of the NLuc/furimazine system to detect light emitted from deep tissues as discussed above.

Although we were able to monitor infection through the chronic phase by detecting circulating parasite biomarkers in serum (S3 Fig), bioluminescence imaging allowed us to identify the tissues that were colonized by the parasite. Taken together, our findings on the tissue tropism of the *T*. *cruzi* Colombiana strain not only correlate with data generated by others [9, 32, 40], but also suggest new potential sites of persistence (e.g. lungs, gut mesenteric fat), demonstrating the parasite’s ability to invade and persist in a wide variety of cell types. Although our studies contribute to a better understanding of CD pathogenesis, more experiments will be required to determine if *T*. *cruzi* Colombiana can persist in additional organs/tissues (e. g. reproductive tract or skin) that were not evaluated in the present work. Within this context, Capewell et al. demonstrated the role of the skin in harboring and transmitting vector-borne African trypanosomes [50]. Likewise, it would be interesting to explore whether the skin constitutes a reservoir of *T*. *cruzi* parasites with a potential role in transmission. Yet, the parasite load in the skin is expected to be very low since we did not detect obvious signal associated with the skin in our vivo imaging studies. Additionally, monitoring the infection for extended periods of time (>126 days) may help to investigate the progression of late chronic disease development.

In conclusion, here we illustrate the functionality of the NLuc- based imaging system as a new tool to study *T*. *cruzi* chronic infections in mice. In addition, the Tc-COL-NLuc/Swiss Webster mouse model may potentially help to achieve a full understanding of long-term parasite persistence, and could be useful to assess drug efficacy against a known chemotherapy resistant *T*. *cruzi* strain.

## Supporting information

S1 Fig Biolumenscence is primarily detected in the abdominal cavity after intravenous or intraperitoneal furimazine delivery. (A) Representative ventral view images of TcCOL-NLuc infected mice (M1 and M2) taken after injection of furimazine (100 μl, 1/10 dilution of Nano-Glo^®^ Luciferase substrate) in the peritoneal cavity (top panel) or via the tail vein (bottom panel). At 21 dpi, animals were injected with the substrate, anesthetized and next transferred to the IVIS 200 system for imaging. (B) Bioluminescence signal (total flux) measured for each animal expressed as total flux (photons/sec). Data represent the median with interquartile range. Grey lines indicate the detection threshold determined as the median (solid line) and median with interquartile range (dashed lines) of background luminescence of the control uninfected mouse. Statistical differences between groups were calculated using an unpaired t-test with a 95% confidence interval in GraphPad Prism. (C) Mice were sacrificed on day 22 post-infection. The hearts were excised and transferred to a 12-well plate. After rinsing with PBS, samples were soaked in a furimazine solution for 5 minutes. Images were acquired using an exposure time of 30 seconds. Log _10_ heat-map scales represent bioluminescence intensity (blue: low; red: high). NI: non-infected control mouse; ns: no significant differences.
(TIF)

S2 Fig Chronic *T*. *cruzi* infection in Swiss Webster mice could be monitored for up to 222 days by live bioluminescence imaging. (A) Ventral view images of mice infected with TcCOL-NLuc tissue culture-derived trypomastigotes (5 x10^3^ parasites) taken at the indicated time points. Log _10_ heat-map scales represent bioluminescence intensity (blue: low; red: high). NI: non-infected control mouse. (B) Bioluminescence signal (total flux) measured at 181 and 222 dpi in the regions of interest indicated by red rectangles in A.
(TIF)

S3 Fig Apt-29 Enzyme Linked Aptamer (ELA) assay detects biomarkers in plasma of TcCOL-NLuc infected mice. Plasma, at 1/200 dilution, from infected mice at different times (21 to 124) post-infection, was coated on a polystyrene 96 well plate. After blocking, biotinylated Apt-29 was added and incubated for 1 hour at room temperature. Next, plates were extensively washed and a Streptavidin-Alkaline phosphatase conjugate was added. After 30 minutes incubation, plates were washed and binding aptamer was detected using 4-Methyliumbeliferyl Phosphate (4-MUP). Fluorescence was read at 340 nm and emission was recorded at 460 nm. Relative fluorescence units (RFU) are plotted on the Y-axis. Each point represents the mean of duplicate values for each individual mouse. Group means and standard deviations are shown. Statistical differences between the ELA signal at each time point with that of non-infected mice were determined using an unpaired t-test with a 95% confidence interval (* *p* < 0.05; ** *p* < 0.01; ns: no significant differences). NI: non-infected mouse.
(TIF)

S4 Fig Standard curve generated with DNA extracted from the heart of a non-infected mouse spiked with TcCOL-NLuc trypomastigotes. The 10-fold dilutions series of spiked heart DNA was amplified using *T*. *cruzi* specific primers. Results are expressed as parasite equivalents in 50 ng of heart DNA and represent the average of triplicate wells. E: efficiency; Ct: cycle threshold.
(TIF)

S1 Table Statistical analysis to compare the fold change radiance among tissues of chronically TcCOL-NLuc-infected mice at 126 days post-infection. Fold-change values in bioluminescence intensity calculated in Fig 5A were further evaluated using a one-way ANOVA analysis. Data represent *p*-values obtained after comparing all tissues. The underlined numbers indicate significantly different values (*p* < 0.05).
(DOC)

S2 Table Statistical analysis to compare parasite loads among tissues of chronically TcCOL-NLuc-infected mice at 126 days post-infection. Parasite loads calculated in Fig 5B were further evaluated using a one-way ANOVA analysis. Data represent *p*-values obtained after comparing all tissues. The underlined numbers indicate significantly different values (*p* < 0.05).
(DOC)
