# Supplementary figures and images for: A novel nanoluciferase-based system to monitor Trypanosoma cruzi infection in mice by bioluminescence imaging
Source: PLoS One. 2018 Apr 19;13(4):e0195879. doi: 10.1371/journal.pone.0195879 (PMC5908157; doi:10.1371/journal.pone.0195879)

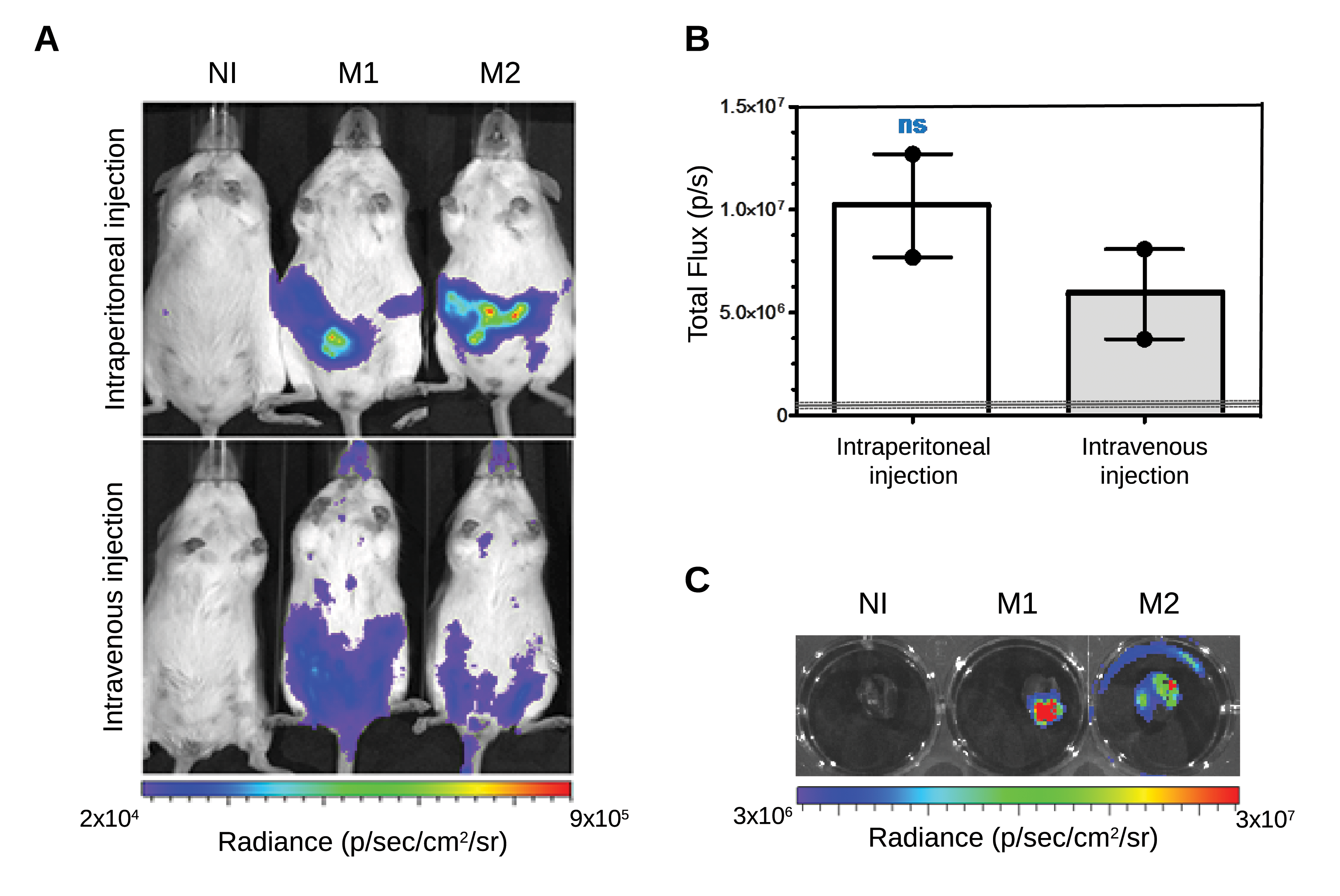

Supplement: S1 Fig — (A) Representative ventral view images of TcCOL-NLuc infected mice (M1 and M2) taken after injection of furimazine (100 μl, 1/10 dilution of Nano-Glo® Luciferase substrate) in the peritoneal cavity (top panel) or via the tail vein (bottom panel). At 21 dpi, animals were injected with the substrate, anesthetized and next transferred to the IVIS 200 system for imaging. (B) Bioluminescence signal (total flux) measured for each animal expressed as total flux (photons/sec). Data represent the median with interquartile range. Grey lines indicate the detection threshold determined as the median (solid line) and median with interquartile range (dashed lines) of background luminescence of the control uninfected mouse. Statistical differences between groups were calculated using an unpaired t-test with a 95% confidence interval in GraphPad Prism. (C) Mice were sacrificed on day 22 post-infection. The hearts were excised and transferred to a 12-well plate. After rinsing with PBS, samples were soaked in a furimazine solution for 5 minutes. Images were acquired using an exposure time of 30 seconds. Log 10 heat-map scales represent bioluminescence intensity (blue: low; red: high). NI: non-infected control mouse; ns: no significant differences. (TIF) [file pone.0195879.s001.tif]

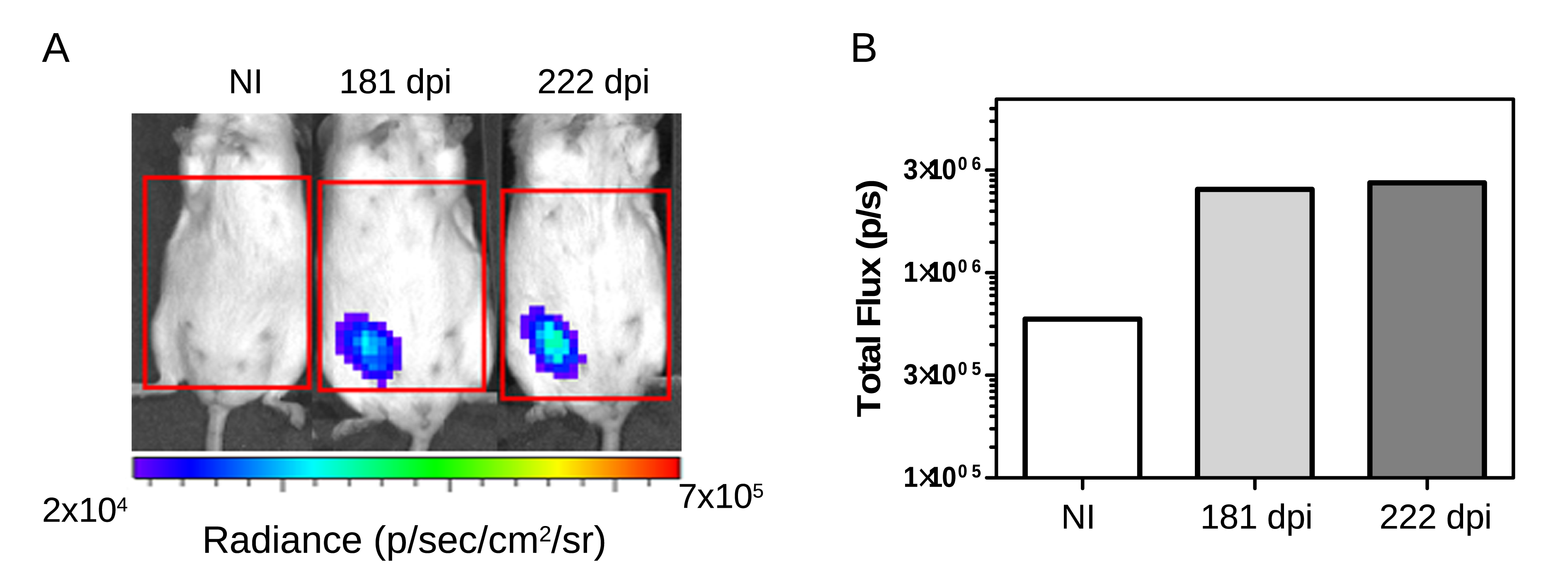

Supplement: S2 Fig — (A) Ventral view images of mice infected with TcCOL-NLuc tissue culture-derived trypomastigotes (5 x10^3^ parasites) taken at the indicated time points. Log 10 heat-map scales represent bioluminescence intensity (blue: low; red: high). NI: non-infected control mouse. (B) Bioluminescence signal (total flux) measured at 181 and 222 dpi in the regions of interest indicated by red rectangles in A. (TIF) [file pone.0195879.s002.tif]

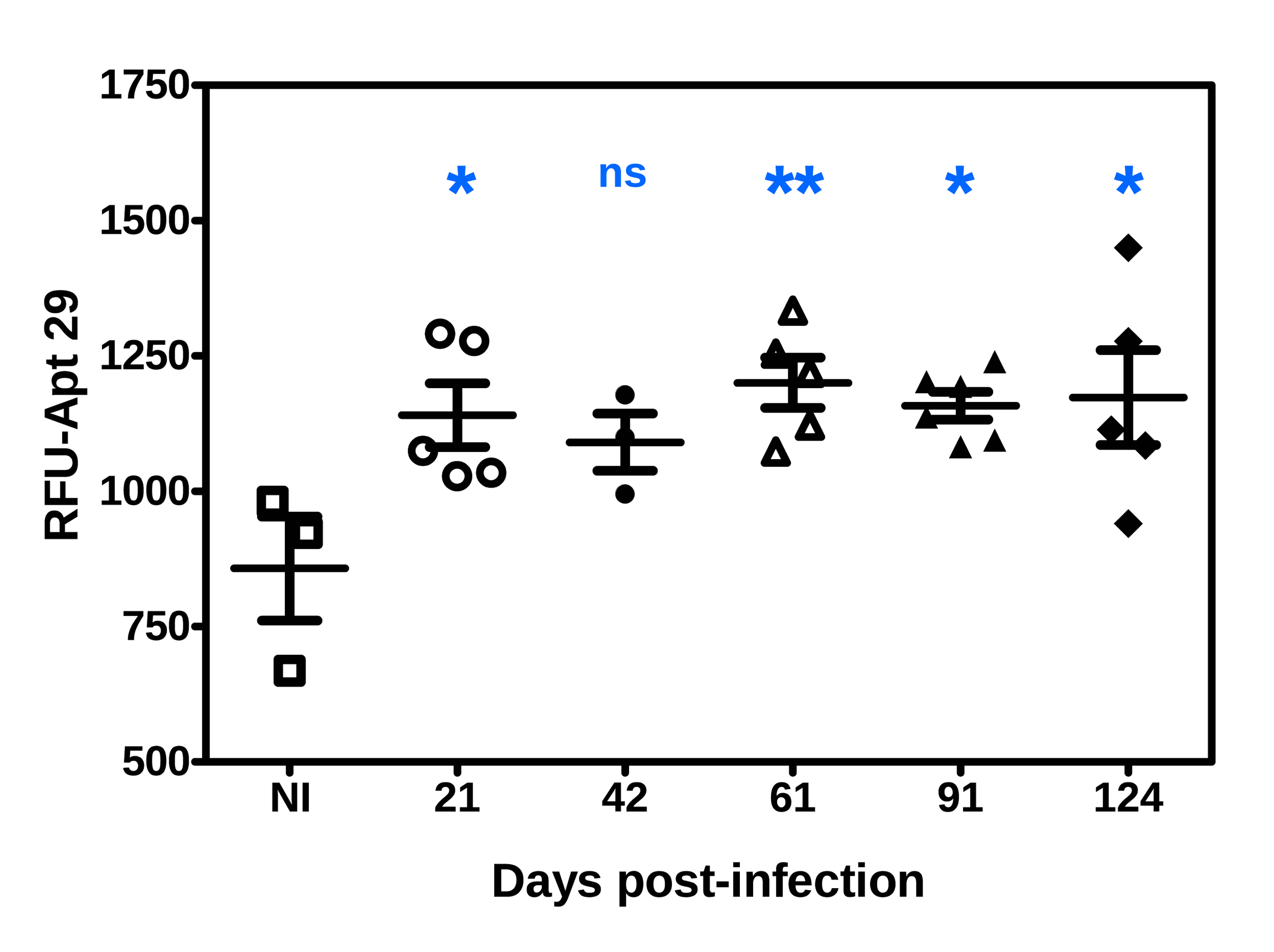

Supplement: S3 Fig — Plasma, at 1/200 dilution, from infected mice at different times (21 to 124) post-infection, was coated on a polystyrene 96 well plate. After blocking, biotinylated Apt-29 was added and incubated for 1 hour at room temperature. Next, plates were extensively washed and a Streptavidin-Alkaline phosphatase conjugate was added. After 30 minutes incubation, plates were washed and binding aptamer was detected using 4-Methyliumbeliferyl Phosphate (4-MUP). Fluorescence was read at 340 nm and emission was recorded at 460 nm. Relative fluorescence units (RFU) are plotted on the Y-axis. Each point represents the mean of duplicate values for each individual mouse. Group means and standard deviations are shown. Statistical differences between the ELA signal at each time point with that of non-infected mice were determined using an unpaired t-test with a 95% confidence interval (* p < 0.05; ** p < 0.01; ns: no significant differences). NI: non-infected mouse. (TIF) [file pone.0195879.s003.tif]

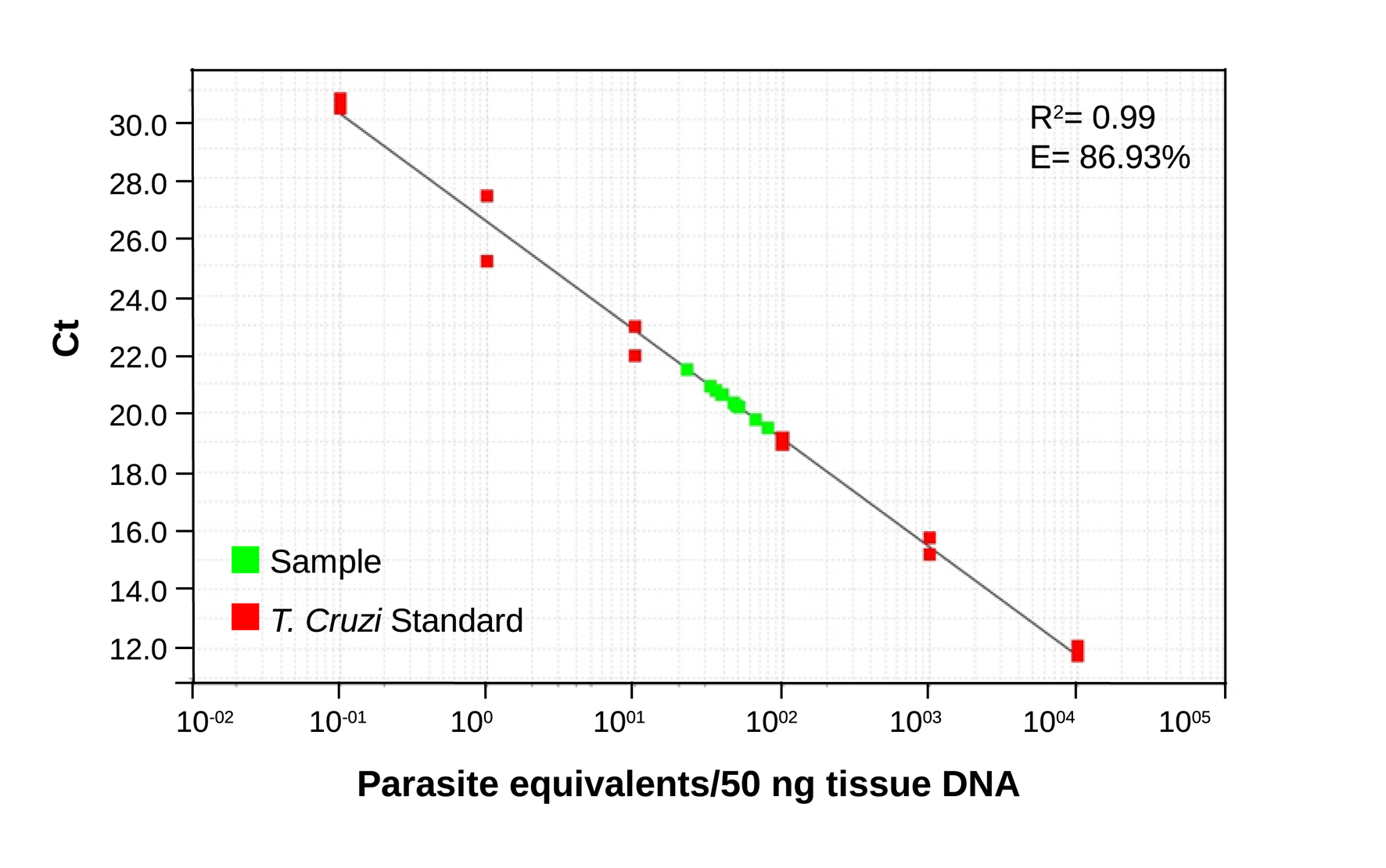

Supplement: S4 Fig — The 10-fold dilutions series of spiked heart DNA was amplified using T. cruzi specific primers. Results are expressed as parasite equivalents in 50 ng of heart DNA and represent the average of triplicate wells. E: efficiency; Ct: cycle threshold. (TIF) [file pone.0195879.s004.tif]
