# Supplementary material for: A novel nanoluciferase-based system to monitor Trypanosoma cruzi infection in mice by bioluminescence imaging
Source: PLoS One. 2018 Apr 19;13(4):e0195879. doi: 10.1371/journal.pone.0195879 (PMC5908157; doi:10.1371/journal.pone.0195879)
Supplement: S1 Table — Fold-change values in bioluminescence intensity calculated in Fig 5A were further evaluated using a one-way ANOVA analysis. Data represent p-values obtained after comparing all tissues. The underlined numbers indicate significantly different values (p < 0.05). (DOC) [file pone.0195879.s005.doc]

**S1 Table. Statistical analysis to compare the fold change radiance among tissues of chronically TcCOL-NLuc-infected mice at 126 days post-infection.**

|  | **Skl** | **M. Fat** | **Lung** | **Liver** | **Intestine** | **V. Fat** | **Spleen** |
| --- | --- | --- | --- | --- | --- | --- | --- |
| **Heart** | 0.6578 | 0.5811 | 0.0791 | **0.0216** | 0.4648 | **0.0121** | **0.0131** |
| **Skl** |  | 0.5175 | 0.3121 | 0.2408 | 0.5024 | 0.2066 | 0.2114 |
| **M. Fat** |  |  | 0.2973 | 0.1159 | 0.9953 | 0.0689 | 0.0744 |
| **Lung** |  |  |  | 0.2569 | 0.1204 | 0.0819 | 0.0972 |
| **Liver** |  |  |  |  | **0.0134** | **0.0386** | 0.0731 |
| **Intestine** |  |  |  |  |  | **0.0052** | **0.0059** |
| **V. Fat** |  |  |  |  |  |  | 0.3372 |

Skl: skeletal muscle; M. Fat: mesenteric fat; V. Fat: visceral fat.

*P*-values were calculated using one-way ANOVA analysis.

***P-*value**: *p* < 0.05; *P-*value*:* *p* > 0.05.
