# Supplementary material for: A novel nanoluciferase-based system to monitor Trypanosoma cruzi infection in mice by bioluminescence imaging
Source: PLoS One. 2018 Apr 19;13(4):e0195879. doi: 10.1371/journal.pone.0195879 (PMC5908157; doi:10.1371/journal.pone.0195879)
Supplement: S2 Table — Parasite loads calculated in Fig 5B were further evaluated using a one-way ANOVA analysis. Data represent p-values obtained after comparing all tissues. The underlined numbers indicate significantly different values (p < 0.05). (DOC) [file pone.0195879.s006.doc]

**S2 Table. Statistical analysis to compare parasite loads among tissues of chronically TcCOL-NLuc-infected mice at 126 days post-infection.**

|  | **Skl** | **M. Fat** | **Lung** | **Liver** | **I R1** | **I R2** | **I R3** | **V. Fat** | **Spleen** |
| --- | --- | --- | --- | --- | --- | --- | --- | --- | --- |
| **Heart** | 0.8740 | **0.0043** | **0.0033** | **0.0030** | **0.0027** | **0.0027** | **0.0026** | **0.0026** | **0.0026** |
| **Skl** |  | 0.3361 | 0.3237 | 0.3120 | 0.3046 | 0.3028 | 0.3003 | 0.3513 | 0.2988 |
| **M. Fat** |  |  | 0.7332 | 0.4859 | 0.3502 | 0.3213 | 0.2866 | 0.2900 | 0.2674 |
| **Lung** |  |  |  | 0.3025 | 0.0582 | **0.0334** | **0.0197** | **0.0203** | **0.0145** |
| **Liver** |  |  |  |  | 0.3886 | 0.2596 | 0.1557 | 0.1625 | 0.1130 |
| **I R1** |  |  |  |  |  | 0.6740 | 0.2956 | 0.3174 | 0.1559 |
| **I R2** |  |  |  |  |  |  | 0.3314 | 0.3649 | 0.1012 |
| **I R3** |  |  |  |  |  |  |  | 0.8735 | 0.2942 |
| **V. Fat** |  |  |  |  |  |  |  |  | 0.1698 |

Skl: skeletal muscle; M. Fat: mesenteric fat; I R1-3: intestine region 1-3; V. Fat: visceral fat

*P*-values were calculated using one-way ANOVA analysis.

***P*-value**: *p* < 0.05; *P*-value*:* *p* > 0.05.
